# Association Between Educational Inequality and Income Inequality With Metabolic Diseases and Cause‐Specific Mortality

**DOI:** 10.1002/clc.70173

**Published:** 2025-07-06

**Authors:** Jingya Niu, Xiaotong Li, Qiaoyun Chen, Wei Yang, Lixia Suo, Zhu Chen

**Affiliations:** ^1^ Jiading District Central Hospital Affiliated Shanghai University of Medicine and Health Sciences Shanghai China; ^2^ School of Clinical Medicine Shanghai University of Medicine and Health Sciences Shanghai China; ^3^ Guangzhou Center for Disease Control and Prevention Guangdong China; ^4^ Xuhang Community Health Service Center, Jiading District Shanghai China

**Keywords:** cardiovascular diseases, education, mortality, population‐attributable fractions, socioeconomic status

## Abstract

**Background:**

Educational attainment and economic status are important socioeconomic characteristics and are associated with metabolic diseases and premature death risk. However, their relative importance and contributions to premature death remain unclear.

**Methods:**

Data were collected from ten survey waves of the National Health and Nutrition Examination Survey from 1999 to 2018. Deaths before age 75 from all‐cause and cause‐specific mortality were ascertained from linkage to the National Death Index with follow‐up through 2019. Weighted Cox proportional hazard models were used to estimate the hazard ratios (HRs) and 95% confidence intervals (CI) for death by educational attainment and income level. Population‐attributable fractions (PAFs) were calculated to quantify the proportional contributions of low income and low educational attainment to mortality.

**Results:**

Over an average of 10.1 years of follow‐up, 4310 premature deaths were confirmed from 43 637 participants. Low income and low educational attainment were associated with increased risks of all‐cause and cause‐specific mortality, respectively. The associations between low educational attainment and mortality risk disappeared after mutual adjusting for income and education. However, among those with high school education or above, the adjusted HRs of middle income and low income were 1.81 (95% CI, 1.48–2.21) and 2.88 (95% CI, 2.31–3.59) for all‐cause mortality. The PAF showed that low educational attainment did not contribute to mortality, while 33.0% of premature deaths were attributable to low income.

**Conclusions:**

Income had a greater impact on mortality risk than education. The disparities in mortality risk could be reduced by narrowing the income differentials.

## Graphical abstract

Low educational attainment and low income are both associated with increased risks of premature mortality. However, income was more important in determining premature mortality. One‐third of premature mortality can be attributed to low income, the disparities in mortality risk could be reduced by narrowing the income differentials.

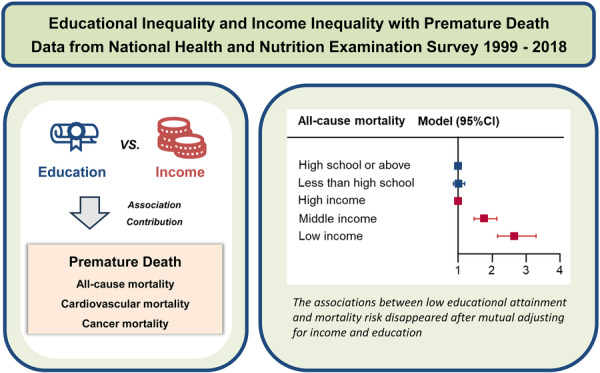

## 1 Introduction

Over the past several decades, socioeconomic progress has substantially improved living conditions in most countries and regions, however, the wealth disparity persist within each nation [1]. Socioeconomic factors comprise a group of heterogeneous yet interrelated elements, but interrelated factors, which cluster in individuals and groups. Low socioeconomic status, including low financial income, limited education, lack of social support, inequality in healthcare access, and poor living conditions, plays an important part in following healthy lifestyles and accessing healthcare. Educational attainment and economic status are the most important socioeconomic characteristics. Both are inversely associated with the risk of premature death, also known as educational inequalities and economic inequalities in mortality [2, 3].

Even in the wealthiest countries, socioeconomic disparities in all‐cause mortality and life expectancy persist in the US population [4, 5]. The burden of cardiovascular disease (CVD)—the leading global cause of morbidity and mortality—varies significantly by socioeconomic status (SES). Several studies have reported that socioeconomic status was associated with cardiovascular diseases (CVD) [6, 7, 8, 9] and mortality [10, 11, 12]. Joshua et al. found that unfavorable social status is associated with increased risks of premature death and contribute to differences between Black and White racial groups in premature all‐cause mortality in the US population [10]. Another study conducted in US population also found that the Black‐White difference in CVD mortality diminished after adjusting for behavioral and metabolic risk factors and completely disappeared with adjusting for social determinants of health [11]. Clinicians could substantially reduce the risks by aggressively managing risk factors in individuals with lower socioeconomic status, and population health managers could target this population for interventions [12].

Educational attainment and income are the most important social determinants of health and both are significantly associated with mortality, however, which factor is more important and contributes more to cardiovascular diseases and deaths remain unclear. The present analysis aims to assess the impact of low educational attainment and low income on the risk of premature mortality and the importance of the two factors. Data from the NHANES (National Health and Nutrition Examination Survey) mortality follow‐up study from 1999 to 2018 were used to achieve these goals.

## 2 Materials & Methods

### 2.1 Study Population

The NHANES is an ongoing, multistage, cross‐sectional survey. This survey uses a complex, stratified, multistage probability sampling design to deliver nationally representative data on the health and nutritional status of the noninstitutionalized civilian population across the United States. Each participant completed a household interview and underwent a physical examination. Detailed descriptions of the plan and operation of each survey have been published (http://www.cdc.gov/nchs/nhanes.htm). The National Center for Health Statistics Research Ethics Review Board reviewed and approved NHANES, and informed consent was obtained for all participants.

The present study is based on an analysis of data from ten survey waves corresponding to NHANES from 1999 to 2018 (*n* = 101 316). According to our aims, participants who were younger than 20 and older than 75 years were excluded (*n* = 53 011). We also excluded the participants without information on educational attainment and household income (*n* = 4311), and those with a follow‐up duration of less than 6 months (*n* = 185). This study followed the Strengthening the Reporting of Observational Studies in Epidemiology guideline for cohort studies.

### 2.2 Independent Variables

The information on educational attainment from participants was collected by self‐reporting the completed highest grade or level of school or the highest received degree. The participants were categorized into five groups, including less than 9th grade, 9th–11th grade, high school graduate/GED or equivalent, some college or AA grade, and college graduate or above. In the present study, less than 9th grade and 9th–11th grade were regarded as low educational attainment [10].

The family poverty‐to‐income ratio (PIR) was used as a proxy of socioeconomic status in our study, which was formulated according to the Department of Health and Human Services (HHS) poverty guidelines (https://aspe.hhs.gov/poverty-guidelines). The HHS poverty guidelines are issued each year, in the Federal Register, to determine financial eligibility for certain federal programs such as Head Start, Supplemental Nutrition Assistance Program (SNAP) (formerly Food Stamp Program), Special Supplemental Nutrition Program for Women, Infants, and Children (WIC), and the National School Lunch Program (https://wwwn.cdc.gov/nchs/nhanes/default.aspx).

The value of PIR was calculated by dividing family income by the poverty guidelines, specific to family size, as well as the appropriate year and state. The values were not computed if the participant did not know the total combined family income. If family income was reported as a range value, the midpoint of the range was used to compute the variable. The PIR values at or above 5.00 were coded as 5.00 or more because of disclosure concerns. The socioeconomic status was classified into three groups: low income (PIR < 1.3), middle income (PIR = 1.3–3.5), and high income (PIR ≥ 3.5) [13].

### 2.3 Assessment of Covariates

Details on the data collection are described on the website (https://wwwn.cdc.gov/nchs/nhanes/analyticguidelines.aspx). During the in‐home interview, age, gender, marriage status, personal behaviors and medical history for diabetes, hypertension, and other conditions were gathered using a standard questionnaire. Current smoking was defined as having smoked at least 100 cigarettes in life and smoking at present. Current alcohol drinking was defined as taking at least 12 times drinks of any type of alcoholic beverage in the last 12 months. Hypertension was defined as systolic blood pressure ≥ 130 mmHg, diastolic blood pressure ≥ 80 mmHg, or a self‐reported previous diagnosis by physician [14]. Diabetes was defined as anyone or a combination of fasting plasma glucose ≥ 7.0 mmol/L, 2‐h post‐load glucose ≥ 11.1 mmol/L, HbA1c ≥ 6.5% (48 mmol/mol), or a self‐reported previous diagnosis by physician. Dyslipidemia is classified as serum total cholesterol, low‐density lipoprotein cholesterol, triglycerides, concentrations above the 90th percentile, or high‐density lipoprotein cholesterol concentrations below the 10th percentile for the general population. The estimated glomerular filtration rate (eGFR) was calculated using the 2009 chronic kidney disease epidemiology collaboration equation with considering the sex, serum creatinine level, and race [15]. Urinary albumin creatinine ratio (UACR) was calculated using urinary albumin divided by urinary creatinine based on morning spot urine. Chronic kidney disease was defined as eGFR level < 60 mL/min/1.73 m^2^ or UACR ≥ 30 mg/g or self‐reported previous diagnosis by physician. Equations expressed for specified sex and serum creatinine level were showed in the Supporting Information S1: Table 1.

### 2.4 Assessment of Outcomes

To examine the associations of educational attainment and income level in relation to risks of all‐cause and cause‐specific mortality, we used data from the NHANES III Linked Mortality File, in which NHANES III‐eligible participants were matched, using a probabilistic matching algorithm, to the National Death Index through December 31, 2019, to determine their mortality status (https://www.cdc.gov/nchs/data-linkage/mortality-public.htm). According to our aims, we evaluated premature death, defined as death occurring before 75 years of age, which was the approximate mean age of death of US adults throughout the study period [16]. We also investigated the associations of educational and income levels with cardiovascular, cancer‐related, and other causes of death before 75 years of age.

International Classification of Diseases, Tenth Revision codes were used to identify participants for the underlying cause of death. Cardiovascular mortality included death due to heart diseases (I00‐I09, I11, I13, I20‐I51) and cerebrovascular diseases (I60‐I69), while cancer mortality included death due to malignant neoplasms (C00‐C97). Follow‐up of participants continued until death with censoring at the time of death. Participants not matched with a death record were considered alive through the entire follow‐up period. A complete, detailed description of the methodology is available.

### 2.5 Statistical Analysis

The appropriate weights and design factors were invoked in all the analyses to account for the multistage probability sampling design of the survey. Demographic and other characteristics of study participants were described in means (95% confidence intervals [CI]) for continuous variables and percentages (95% CI) for categorical variables.

The associations between educational attainment and income level with metabolic diseases were assessed using logistic regression. The weighted Kaplan‐Meier curves were used to present the rate of all‐cause and cause‐specific mortality. Survival rates by groups were compared using the Log‐rank test. The survival probabilities were estimated as the time intervals from the date of the interview to the last follow‐up time, December 31, 2019, or the date of death. Cox proportional hazard models were used to estimate the hazard ratios (HRs) and 95% CI of death for educational attainment and income level. We selected pre‐specified potential confounders for adjustment in multivariable models, including age, gender, current smoking (yes/no), current drinking (yes/no), high level of physical activity (yes/no), marriage status (married/not married), BMI, diabetes (yes/no), hypertension (yes/no), dyslipidemia (yes/no) and CKD (yes/no).

Population‐attributable fractions (PAFs) and associated 95% CIs quantified the proportional reduction in mortality that would be achieved if the risk factors were theoretically removed from the population. We used the approach described by Eide and Gefeller [17] and the “averisk” R package to calculate the PAF of mortality due to low educational attainment and low income in one model adjusted for covariates. In this approach, the PAF and 95% CI for each risk factor are determined using logistic regression, all risk factors are added to the model, and the average of all PAFs is then calculated.

In this study, all tests were two‐sided, and the type I error α was set to be 0.05. Statistical analyses were done using R (version 4.1.2). The complexity of the sampling design was considered in each analysis by specifying primary sampling units, strata, and weights using the R package “survey” (version 4.1‐1). We used MEC exam weights for all sample estimations.

## 3 Results

### 3.1 Characteristics of Participants

A total of 43 637 participants were included in the final analysis. The weighted prevalence of participants with less than high school and high school education or above was 16.0% (95% CI = 15.1%–16.8%) and 84.0% (95% CI = 83.2%–84.9%), respectively. The weighted prevalence of participants with low income, middle income and high income were 21.4% (95% CI = 20.3%–22.4%), 34.7% (95% CI = 33.7%–35.7%), and 43.9% (95% CI = 42.4%–45.4%), respectively. Demographic and clinical characteristics of study participants are presented in Table 1. Participants with less than a high school education were characterized by significantly more smokers but fewer drinkers, and worse metabolic profiles than those with higher education levels. Participants with low income were younger, had a higher proportion of unmarried individuals, more smokers and fewer drinkers, and worse metabolic profiles than those with high income. Over an average of 10.1 years of follow‐up, 4310 deaths were confirmed. Among them, 1178 were due to cardiovascular disease, 1154 were due to cancer, and 1978 were due to other causes. The weighted incidences of leading causes of death by educational attainment and income level are shown in Supporting Information S1: Table 2.

**Table 1:** The characteristics of participants by educational attainment and income level.

	Less than high school (*n* = 11 018)	High school or above (*n* = 32 619)	Low income (*n* = 13 572)	Middle income (*n* = 16 058)	High income (*n* = 14 007)
Age (year), mean	44.9 (44.4–45.3)	43.9 (43.6–44.3)	40.7 (40.1–41.3)	43.7 (43.3–44.2)	46.5 (46.0–46.9)
Female, %	48.2 (47.1–49.4)	51.9 (51.4–52.5)	55.9 (55.0–56.8)	51.5 (50.7–52.3)	49.0 (48.3–49.8)
Family income‐to‐poverty ratio, mean	1.80 (1.74–1.86)	3.28 (3.23–3.33)	0.78 (0.77–0.79)	2.33 (2.31–2.35)	4.68 (4.66–4.70)
Currently married, %	50.1 (48.3–51.9)	57.0 (55.7–58.3)	35.6 (34.1–37.2)	52.5 (50.9–54.2)	68.4 (66.9–69.9)
Currently smoking, %	56.9 (55.2–58.6)	44.1 (43.0–45.1)	52.7 (51.0–54.4)	48.6 (47.2–50.0)	40.9 (39.7–42.2)
Currently drinking, %	70.3 (68.9–71.6)	80.3 (79.0–81.5)	71.4 (70.0–72.8)	76.6 (75.1–78.0)	83.9 (82.7–85.0)
Body mass index (kg/m^2^), mean	29.15 (28.89–29.31)	28.84 (28.65–28.95)	29.23 (29.01–29.39)	29.21 (29.01–29.39)	28.46 (28.32–28.68)
Waist circumference (cm), mean	98.99 (98.50–99.46)	98.17 (97.79–98.55)	98.38 (97.88–98.88)	98.87 (98.38–99.36)	97.81 (97.34–98.28)
Systolic blood pressure (mm Hg), mean	123.7 (122.4–123.6)	120.6 (120.7–121.3)	121.0 (120.5–121.5)	121.5 (120.6– 121.4)	120.8 (120.6–121.4)
Diastolic blood pressure (mm Hg), mean	70.8 (70.4–71.3)	71.8 (71.5–72.1)	70.7 (70.3–71.2)	71.2 (70.8–71.6)	72.4 (72.1–72.7)
HbA1c (%), mean	5.74 (5.71–5.77)	5.50 (5.48–5.52)	5.63 (5.60–5.66)	5.56 (5.54–5.58)	5.48 (5.46–5.50)
Fasting plasma glucose (mmol/L), mean	6.07 (6.00–6.15)	5.74 (5.71–5.78)	5.89 (5.84–5.95)	5.83 (5.78–5.89)	5.72 (5.68–5.77)
2 h postprandial glucose (mmol/L), mean	6.59 (6.42–6.76)	6.25 (6.17–6.33)	6.43 (6.28–6.58)	6.33 (6.23–6.44)	6.21 (6.10–6.33)
Triglyceride (mmol/L), mean	1.73 (1.68–1.80)	1.54 (1.52–1.57)	1.64 (1.59–1.68)	1.60 (1.56–1.64)	1.52 (1.49–1.56)
HDL‐cholesterol (mmol/L), mean	1.29 (1.28–1.31)	1.38 (1.37–1.39)	1.31 (1.30–1.32)	1.35 (1.34–1.36)	1.42 (1.41–1.43)
Total cholesterol (mmol/L), mean	5.10 (5.07–5.14)	5.08 (5.06–5.10)	4.99 (4.96–5.02)	5.07 (5.04–5.10)	5.14 (5.11–5.17)
LDL‐cholesterol (mmol/L), mean	3.05 (3.02–3.10)	3.02 (2.99–3.05)	2.97 (2.93–3.00)	3.00 (2.97–3.04)	3.03 (3.01–3.06)
Urinary albumin‐creatinine ratio (mg/g)	21.10 (20.05–22.15)	14.91 (14.40–15.43)	20.72 (19.5–21.88)	17.01 (16.25–17.77)	12.67 (12.01–13.33)
Estimated glomerular filtration rate (ml/min/1.73 m^2^)	111.98 (111.33–112.63)	109.44 (108.97–109.91)	115.69 (114.96–116.43)	110.94 (110.39–111.49)	106.19 (105.71–106.66)
Taking insulin, %	3.1 (2.7–3.5)	2.0 (1.8–2.2)	3.0 (2.7–3.3)	2.3 (2.0–2.6)	1.7 (1.4–2.0)
Taking oral hypoglycemic agents, %	5.6 (5.3–5.9)	4.1 (3.9–4.3)	4.6 (4.4–4.9)	4.9 (4.6–5.1)	3.9 (3.6–4.1)
Taking antihypertensive agents, %	25.4 (24.1–26.7)	21.7 (21.0–22.4)	22.2 (21.1–23.4)	22.8 (21.8–23.8)	21.8 (20.9–22.8)
Taking lipid‐lowering agents, %	13.5 (12.4–14.5)	12.9 (12.4–13.4)	10.7 (9.91–11.6)	12.3 (11.6–13.1)	14.6 (13.8–15.4)

### 3.2 Associations of Educational Inequality and Income Inequality With Metabolic Diseases and Cause‐Specific Mortality

**Figure 1:**
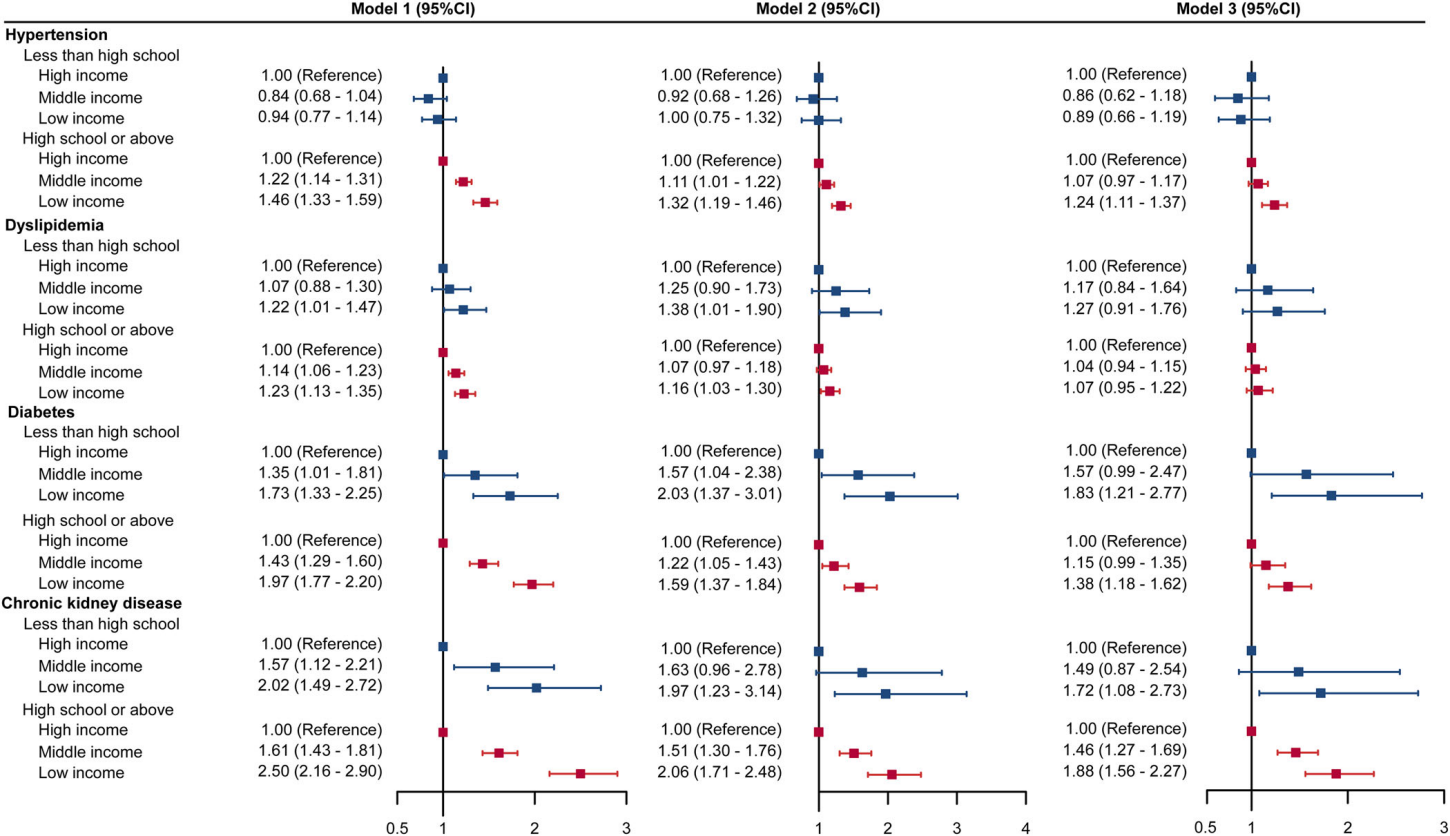
Odds ratios of metabolic diseases associated with income level categories by educational attainment. Model 1 was adjusted for age and gender. Model 2 was additionally adjusted for smoking status, drinking status, marriage status, and body mass index. Model 3 was additionally mutually adjusted for metabolic diseases, including hypertension, hyperlipidemia, diabetes, and chronic kidney diseases.

**Figure 2:**
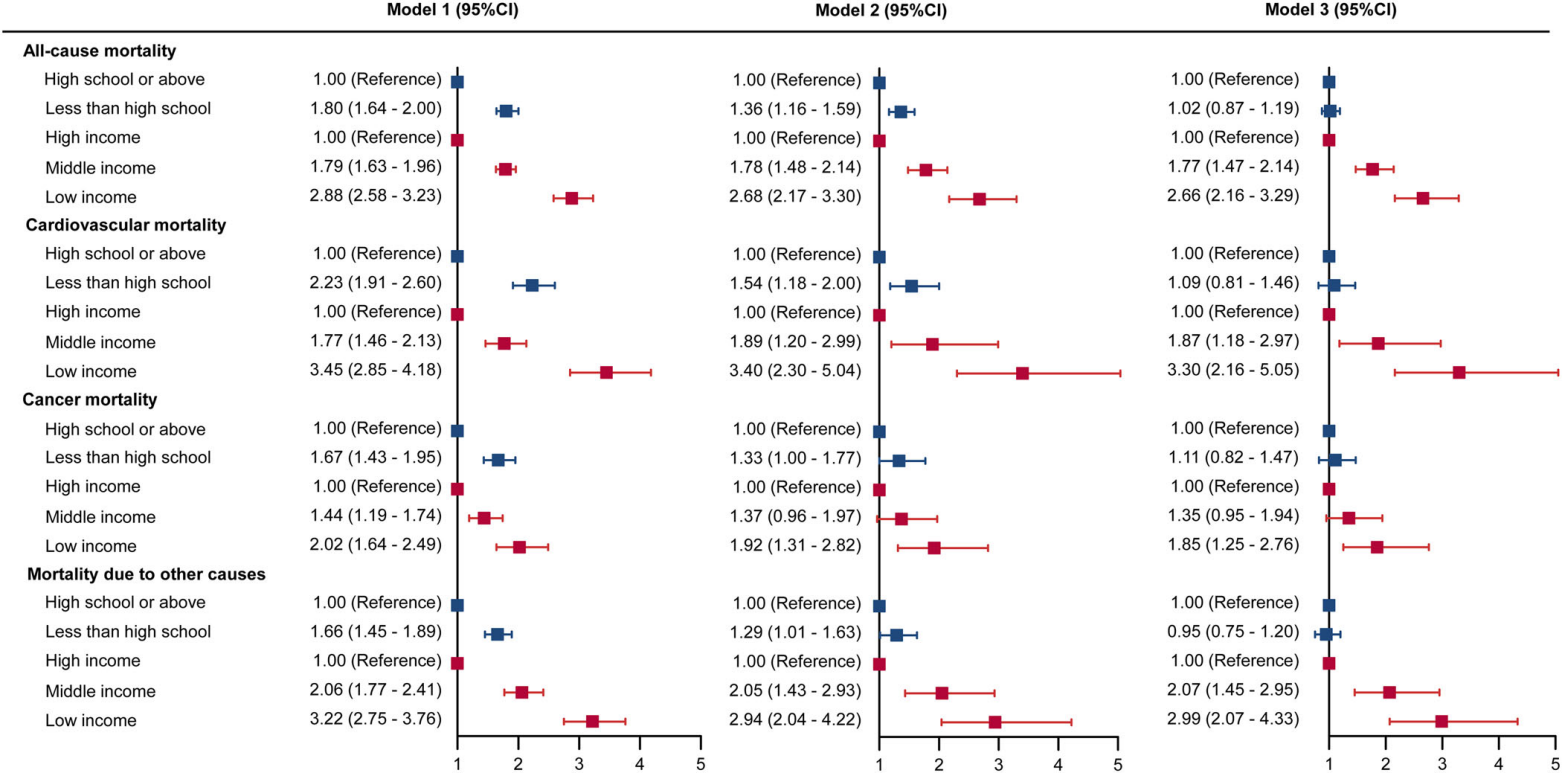
Hazard ratios of cause‐specific mortality associated with educational attainment and income level categories, respectively. In model 1, the estimate of hazard ratios of low educational attainment and low income was conducted in separate model with adjustment of age and gender. In model 2, the estimate of hazard ratios of low educational attainment and low income was conducted in separate model with adjustment of age, gender, smoking status, drinking status, marriage status, body mass index, hypertension, hyperlipidemia, diabetes, and chronic kidney diseases. In model 3, the estimate of hazard ratios of low educational attainment and low income was conducted in one model with adjustment of age, gender, smoking status, drinking status, marriage status, body mass index, hypertension, hyperlipidemia, diabetes, and chronic kidney diseases.

Among participants who had less than a high school education, the mortality rates per 1000 person‐years for all‐cause mortality, cardiovascular mortality, cancer mortality, and mortality due to other causes were significantly higher than those with a high school education or above (Supporting Information S1: Table 3). Similarly, the mortality rates were stepwise increased from those with high income to low income (Supporting Information S1: Table 3). The Kaplan‐Meier curves for all‐cause, cardiovascular mortality, cancer mortality, and mortality due to other causes by education level and income level are presented in Supporting Information S1: Figures 1–2, respectively (all log‐rank *p* < 0.001).

We observe significant differences in the associations between income levels with metabolic diseases by educational attainment (Figure 1). Among the participants with less than high school education, the associations between low income with hypertension and dyslipidemia were not significant in fully adjusted model 3. However, there were significant associations between income levels with prevalent diabetes and CKD for participants with different educational attainments (Figure 1).

Comparing participants with high school education or above, the adjusted HR was 1.36 (95% CI, 1.16–1.59) for all‐cause mortality, 1.54 (95% CI, 1.18–2.00) for cardiovascular mortality, 1.33 (95% CI, 1.00–1.77) for cancer mortality, and 1.29 (95% CI, 1.01–1.63) for mortality due to other causes in model 2 (Figure 2). Comparing participants with high income, those with middle income and low income also presented significantly higher mortality risk in model 2 (Figure 2). However, in model 3, we assessed the mortality risk of different educational attainment and income levels in one model, and the associations between low educational attainment and mortality risk completely vanished, while middle income and low income remained significantly higher risks of mortality compared with high income group (Figure 2).

**Figure 3:**
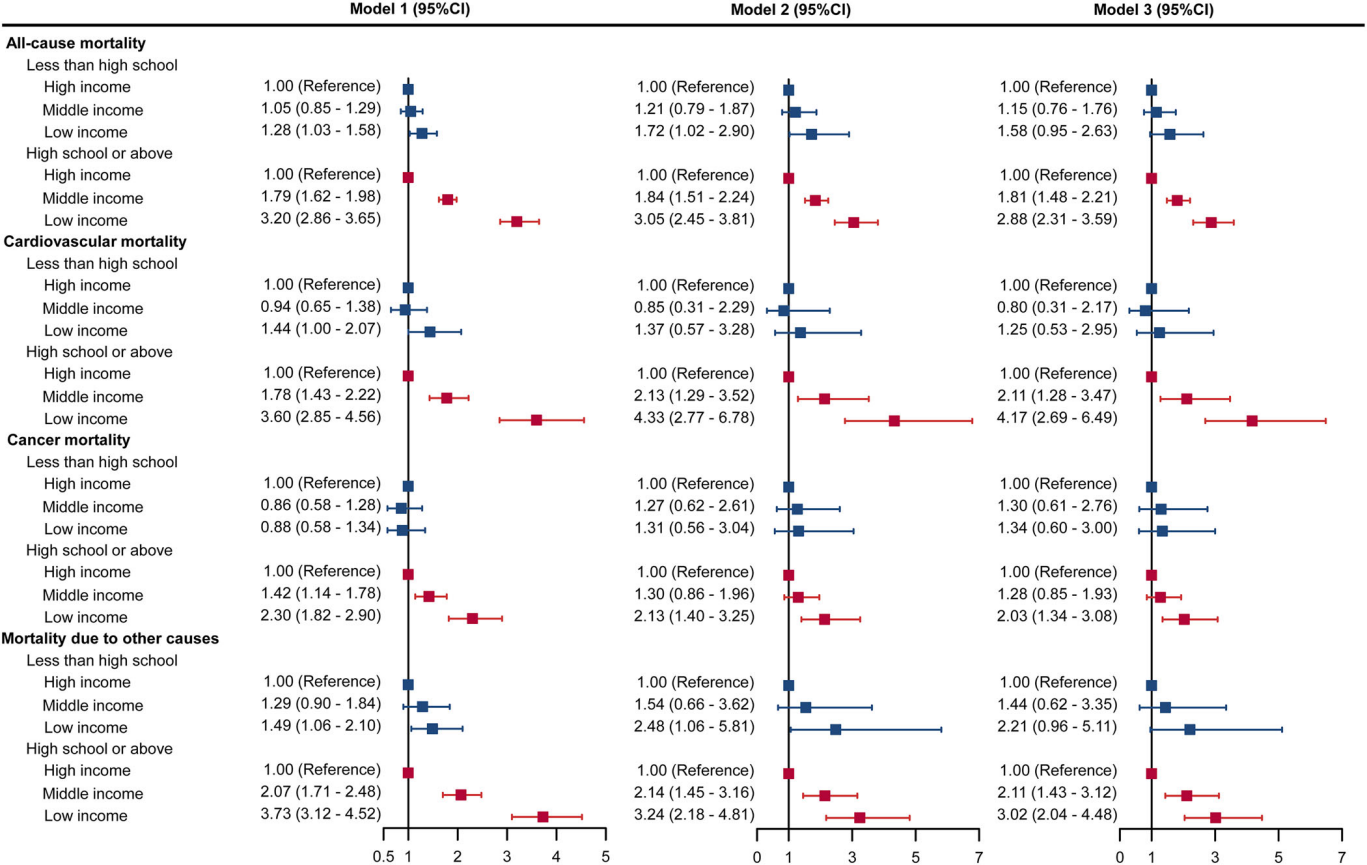
Hazard ratios of cause‐specific mortality associated with income level categories by educational attainment. Model 1 was adjusted for age and gender. Model 2 was additionally adjusted for smoking status, drinking status, marriage status and body mass index. Model 3 was additionally adjusted for hypertension, hyperlipidemia, diabetes, and chronic kidney diseases.

Then, we categorized the participants into two groups by educational attainment. Among participants with high school education or above, the mortality rates were stepwise increased from those with high income to low income (all *p* for trend < 0.001) (Supporting Information S1: Table 4). In contrast, the trends of mortality rates by income level were not evident among those with less than a high school education (Supporting Information S1: Table 4). As shown in Figure 3, among the participants with less than a high school education, the income levels were not significantly associated with increased risk of all‐cause and cause‐specific mortality. However, among those with a high school education or above, both middle‐income (adjusted HR 1.81, 95% CI: 1.48–2.21) and low‐income (adjusted HR 2.88, 95% CI: 2.31–3.59) groups had significantly elevated risks of all‐cause mortality compared to high‐income participants. Similar results were also observed for cardiovascular mortality, cancer mortality and mortality due to other causes (Figure 3).

### 3.3 PAFs of Low Educational Attainment and Low Income for All‐Cause and Cause‐Specific Mortality

We evaluated the PAFs for low educational attainment and low income using three models for all‐cause and cause‐specific mortality (Table 2). In model 1, the estimates of low educational attainment and low income were performed in a separate model with adjustment of age and gender, both the two factors significantly contributed to all‐cause and cause‐specific mortality. After adjusting for other covariates in model 2, low educational attainment did not significantly contribute to cancer mortality. However, when the estimates of PAFs of low educational attainment and low income were conducted in one model, the low educational attainment did not significantly contribute to all‐cause and cause‐specific mortality in model 3. Adjusted PAFs of low income were 33.0% (24.7%–40.3%) for all‐cause mortality, 41.7% (25.2%–58.2%) for cardiovascular mortality, 21.1% (5.9–35.8%) for cancer mortality, and 37.2% (24.9%–50.0%) for mortality due to other causes.

**Table 2:** The population‐attributable fraction of low educational attainment and low income for all‐cause and cause‐specific mortality.

	All‐cause mortality	Cardiovascular mortality	Cancer mortality	Mortality due to other causes
Model 1				
Less than high school	16.3% (13.1%–18.9%)	21.9% (17.1%–27.7%)	10.8% (5.1%–17.3%)	14.9% (10.7%–19.8%)
Low income	36.2% (32.8%–40.1%)	40.8% (34.0%–48.2%)	23.8% (14.9%–34.2%)	39.8% (34.6%–46.1%)
Model 2				
Less than high school	7.0% (1.1%–13.2%)	9.9% (2.0%–19.3%)	3.2% (−9.8%–16.4%)	9.0% (1.1%–16.8%)
Low income	35.3% (27.2%–44.3%)	45.1% (27.8%–62.9%)	20.8% (3.2%–38.6%)	41.2% (29.3%–52.4%)
Model 3				
Less than high school	2.9% (−0.9%– 7.0%)	4.8% (−3.0%–11.6%)	1.1% (−5.0%–6.8%)	4.0% (−1.1%–9.4%)
Low income	33.0% (24.7%–40.3%)	41.7% (25.2%–58.2%)	21.1% (5.9%–35.8%)	37.2% (24.9%–50.0%)

*Note:* Data are expressed as percentage and 95% CI. In model 1, the estimates of population‐attributable fraction of low educational attainment and low income was conducted in separate model with adjustment of age and gender. In model 2, the estimates of population‐attributable fraction of low educational attainment and low income was conducted in separate model with adjustment of age, gender, smoking status, drinking status, marriage status, BMI, hypertension, hyperlipidemia, diabetes, and chronic kidney disease. In model 3, the estimates of population‐attributable fraction of low educational attainment and low income was conducted in one model with adjustment of age, gender, smoking status, drinking status, marriage status, BMI, hypertension, hyperlipidemia, diabetes, and chronic kidney disease.

## 4 Discussion

This study assessed the importance of educational inequalities and economic inequalities on the risk of premature mortality. Low educational attainment and low income were strongly associated with increased risks of premature mortality, respectively. However, after mutually adjusting for education and income levels, there was no significant association between education levels with premature mortality. One‐third of premature mortality can be attributed to low income, but none to low education if the contributions of the two factors to mortality were assessed with mutual adjustment. Income had a greater impact on mortality risk than education.

Our results are consistent with data from other studies that showed higher inequality in overall mortality due to low income or low educational attainment. In an analysis of data from the UK Biobank, Zhang et al. used data from UK Biobank reported that individuals with low socioeconomic status, such as education level, household income, and employment status, had a 2.25‐fold higher risk for cardiovascular death than those with high socioeconomic status [18]. Generally, a higher level of education is typically associated with better jobs and higher incomes, and have richer resources, better environments, and healthy lifestyles [19]. However, based on our analysis, the association and contribution of low education on mortality diminished to none after adjusting for income level. The PURE (Prospective Urban Rural Epidemiologic) study also explored the association between education and household wealth and cardiovascular disease and mortality. They found that low education was still associated with an increased risk for major cardiovascular diseases and mortality, but household wealth, consistent with measures of income and expenditure, was not associated with outcomes in high‐income countries [20]. PURE study is the largest prospective study collected data from 20 countries. In high‐income countries, the health‐care system could provide potential support for preventing many events by improving the use of simple proven treatments, especially in individuals with lower education level [20]. Nevertheless, as stated in the report, their findings might not be applicable to some countries within a specific economic category‐for example, the US‐where the social and health‐care systems differ substantially from other high‐income countries [20].

Other countries, including New Zealand, Canada, Colombia and many European countries, had also quantified the socioeconomic inequalities in mortality [21, 22, 23]. These studies also found that, compared to people with higher socioeconomic status, those with lower socioeconomic status had elevated mortality rates, regardless of whether their status was determined by education or income. Our estimates of HRs for the lowest compared with the highest education groups or the highest income groups were similar to the most recently published estimates. However, few studies have compared the importance of education and income on the effects of mortality. The results of our study indicated that income was independently associated with all‐cause and cause‐specific mortality after accounting for the effect of education. Similarly, another study based on a large cohort of insurance enrollees in Germany also observed that income overrides the effect of education and occupation on mortality [24]. High income was associated with healthier lifestyles, better living conditions and improved access to medical care. The analyses of PAFs for mortality in our study showed that low income contributed to almost one‐third of premature death after adjusting for other factors. However, the mortality burden was not affected by low educational attainment after accounting for income, which suggested that the educational differences in mortality might partly be mediated through the differences in income.

This study has implications for both clinical care and population health management. Our findings suggest that assessing a person's socioeconomic level may be a practical strategy for identifying high‐risk individuals for targeted intervention. Population health managers could identify these patients to improve linkages to community resources including housing, social support, availability of healthy foods, or transportation, so as to narrow the income differentials and health disparities.

The present study has several strengths, including its nationally representative sample and systematic collection of social, behavioral, and metabolic risk factors using standard methods, along with mortality data linked to national death records. However, there were also limitations. First, the data were collected only once, the changes in income on mortality could not be evaluated. Second, the baseline data of NHANES extended over 20 years from 1999 to 2018, the secular trends might exist. Third, data on incident diseases were not collected, therefore the interpretation is limited to the mortality.

## 5 Conclusion

Inequalities in mortality have increased between groups with different income and educational levels. Low income was associated with increased risk of premature mortality and contributed to one‐third of mortality independent of other demographic, lifestyle and metabolic risk factors. In contrast, education was not associated with or contributed to premature mortality after adjusting for income. Our findings suggested that income was more important in determining premature mortality than education. The disparities of mortality risk could be reduced by narrowing the income differentials.

## Author Contributions

Jingya Niu, Zhu Chen, Wei Yang, Lixia Suo designed the study; Jingya Niu analyzed and validated the data. Xiaotong Li and Qiaoyun Chen interpreted the data. Jingya Niu drafted the manuscript. Zhu Chen, Lixia Suo and Wei Yang revised it. All authors agreed to be accountable for all aspects of the work and approved the paper.

## Ethics Statement

The studies involving human participants were reviewed and approved by Ethical approval for the use of the NHANES survey data from 1999 to 2018 were obtained from the National Center for Health Statistics (NCHS) Research Ethics Review Board (ERB). All participants in this study were provided written informed consent. The information collected by the NCHS was kept with strict confidentiality bound to law. The patients/participants provided their written informed consent to participate in this study.

## Conflicts of Interest

The authors declare no conflicts of interest.

## Supporting information

Supplementary_file.

## Data Availability

The data sets generated and analyzed during the current study are available in the NHANES repository, (https://wwwn.cdc.gov/nchs/nhanes/Default.aspx).
