# Supplementary material for: Association Between Educational Inequality and Income Inequality With Metabolic Diseases and Cause‐Specific Mortality
Source: Clin Cardiol. 2025 Jul 6;48(7):e70173. doi: 10.1002/clc.70173 (PMC12228986; doi:10.1002/clc.70173)
Supplement: Supplementary file 1 — Supplementary_file. [file CLC-48-e70173-s001.docx]

**Supplementary file**

Supplementary Table 1. Equations expressed for specified sex and serum creatinine level

| Gender | Serum creatinine | Equation for estimating GFR |
| --- | --- | --- |
| Female | ≤0.7 mg/dl | 144 ◊ (SCr/0.7)^-0.329^ ◊ 0.993 ^Age^ [◊ 1.159 if black] |
| Female | >0.7 mg/dl | 144 ◊ (SCr/0.7)^-1.209^ ◊ 0.993 ^Age^ [◊ 1.159 if black] |
| Male | ≤0.9 mg/dl | 144 ◊ (SCr/0.9)^-0.411^ ◊ 0.993 ^Age^ [◊ 1.159 if black] |
| Male | >0.9 mg/dl | 144 ◊ (SCr/0.9)^-1.209^ ◊ 0.993 ^Age^ [◊ 1.159 if black] |

Supplementary table 2. The weighted incidence of leading causes of death by education attainment and income level

|  | High school or above | | |  | Less than high school | | |
| --- | --- | --- | --- | --- | --- | --- | --- |
|  | High income | Middle income | Low income |  | High income | Middle income | Low income |
| Diseases of heart (I00-I09, I11, I13, I20-I51) (%) | 0.93 (0.75 - 1.11) | 1.47 (1.23 - 1.71) | 1.91 (1.48 - 2.34) |  | 3.78 (2.53 - 5.03) | 3.10 (2.30 - 3.90) | 3.63 (2.96 - 4.30) |
| Malignant neoplasms (C00-C97) (%) | 1.50 (1.25 - 1.75) | 2.00 (1.67 - 2.33) | 2.00 (1.65 - 2.35) |  | 5.00 (3.31 - 6.69) | 3.80 (3.02 - 4.58) | 3.00 (2.45 - 3.55) |
| Chronic lower respiratory diseases (J40-J47) (%) | 0.23 (0.14 - 0.32) | 0.50 (0.36 - 0.64) | 0.49 (0.33 - 0.65) |  | 0.89 (0.35 - 1.43) | 1.19 (0.79 - 1.59) | 0.80 (0.50 - 1.10) |
| Accidents (unintentional injuries) (V01-X59, Y85-Y86) (%) | 0.25 (0.16 - 0.34) | 0.43 (0.30 - 0.56) | 0.54 (0.31 - 0.77) |  | 0.91 (0.02 - 1.80) | 0.66 (0.25 - 1.07) | 0.58 (0.37 - 0.79) |
| Cerebrovascular diseases (I60-I69) (%) | 0.18 (0.12 - 0.24) | 0.28 (0.17 - 0.39) | 0.27 (0.15 - 0.39) |  | 0.73 (0.14 - 1.32) | 0.67 (0.39 - 0.95) | 0.67 (0.37 - 0.97) |
| Alzheimer's disease (G30) (%) | 0.10 (0.05 - 0.15) | 0.22 (0.13 - 0.31) | 0.09 (0.02 - 0.16) |  | 0.25 (0.00 - 0.61) | 0.34 (0.10 - 0.58) | 0.13 (0.04 - 0.22) |
| Diabetes mellitus (E10-E14) (%) | 0.21 (0.12 - 0.30) | 0.21 (0.11 - 0.31) | 0.33 (0.18 - 0.48) |  | 0.40 (0.20 - 0.60) | 0.89 (0.52 - 1.26) | 0.69 (0.48 - 0.90) |
| Influenza and pneumonia (J09-J18) (%) | 0.06 (0.01 - 0.10) | 0.10 (0.04 - 0.16) | 0.16 (0.06 - 0.25) |  | 0.17 (0.00 - 0.38) | 0.23 (0.05 - 0.40) | 0.18 (0.09 - 0.27) |
| Nephritis, nephrotic syndrome and nephrosis (N00-N07, N17-N19, N25-N27) (%) | 0.06 (0.03 - 0.09) | 0.12 (0.05 - 0.19) | 0.19 (0.09 - 0.29) |  | 0.45 (0.05 - 0.85) | 0.24 (0.05 - 0.43) | 0.36 (0.19 - 0.53) |
| All other causes (residual) (%) | 1.10 (0.88 - 1.32) | 2.20 (1.89 - 2.51) | 2.70 (2.23 - 3.17) |  | 2.50 (1.48 - 3.52) | 2.80 (2.15 - 3.45) | 3.00 (2.39 - 3.61) |

Supplementary Table 3. All-cause and cause-specific mortalities by educational attainment and by income level, respectively

|  | Events, n | Person-years, n | Mortality rate per 1000 person-years (95%CI) |
| --- | --- | --- | --- |
| All-cause mortality |  |  |  |
| High school or above | 2584 | 324946.8 | 8.0 (7.6 - 8.3) |
| Less than high school | 1726 | 117646.8 | 14.7 (14.0 - 15.4) |
| High income | 936 | 149179.5 | 6.3 (5.9 - 6.7) |
| Middle income | 1701 | 162162.1 | 10.5 (10.0 - 11.0) |
| Low income | 1673 | 131252 | 12.8 (12.1 - 13.4) |
| Cardiovascular mortality |  |  |  |
| High school or above | 651 | 324946.8 | 2.0 (1.9 - 2.2) |
| Less than high school | 527 | 117646.8 | 4.5 (4.1 - 4.9) |
| High income | 239 | 149179.5 | 1.6 (1.4 - 1.8) |
| Middle income | 461 | 162162.1 | 2.8 (2.6 - 3.1) |
| Low income | 478 | 131252 | 3.6 (3.3 - 4.0) |
| Cancer mortality |  |  |  |
| High school or above | 732 | 324946.8 | 2.3 (2.1 - 2.4) |
| Less than high school | 422 | 117646.8 | 3.6 (3.3 - 3.9) |
| High income | 298 | 149179.5 | 2.0 (1.8 - 2.2) |
| Middle income | 446 | 162162.1 | 2.8 (2.5 - 3.0) |
| Low income | 410 | 131252 | 3.1 (2.8 - 3.4) |
| Mortality due to other causes |  |  |  |
| High school or above | 1201 | 324946.8 | 3.7 (3.5 - 3.9) |
| Less than high school | 777 | 117646.8 | 6.6 (6.1 - 7.1) |
| High income | 399 | 149179.5 | 2.7 (2.4 - 3.0) |
| Middle income | 794 | 162162.1 | 4.9 (4.6 - 5.2) |
| Low income | 785 | 131252 | 6.0 (5.6 - 6.4) |

Supplementary Table 4. All-cause and cause-specific mortalities by educational attainment and income level

|  | Events, n | Person-years, n | Mortality rate per 1000 person-years (95%CI) |
| --- | --- | --- | --- |
| All-cause mortality |  |  |  |
| Less than high school |  |  |  |
| High income | 177 | 11698 | 15.1 (13.0 - 17.5) |
| Middle income | 634 | 44412 | 14.3 (13.2 - 15.4) |
| Low income | 915 | 61537 | 14.9 (13.9 - 15.9) |
| *P* for linear trend |  |  | 0.679 |
| High school or above |  |  |  |
| High income | 759 | 137482 | 5.5 (5.1 - 5.9) |
| Middle income | 1067 | 117750 | 9.1 (8.5 - 9.6) |
| Low income | 758 | 69715 | 10.9 (10.1 - 11.7) |
| *P* for linear trend |  |  | <0.001 |
| Cardiovascular mortality |  |  |  |
| Less than high school |  |  |  |
| High income | 53 | 11698 | 4.5 (3.4 - 5.9) |
| Middle income | 189 | 44412 | 4.3 (3.7 - 4.9) |
| Low income | 285 | 61537 | 4.6 (4.1 - 5.2) |
| *P* for linear trend |  |  | 0.438 |
| High school or above |  |  |  |
| High income | 186 | 137482 | 1.4 (1.2 - 1.6) |
| Middle income | 272 | 117750 | 2.3 (2.0 - 2.6) |
| Low income | 193 | 69715 | 2.8 (2.4 - 3.2) |
| *P* for linear trend |  |  | <0.001 |
| Cancer mortality |  |  |  |
| Less than high school |  |  |  |
| High income | 50 | 11698 | 4.3 (3.2 - 5.6) |
| Middle income | 158 | 44412 | 3.6 (3.0 - 4.2) |
| Low income | 214 | 61537 | 3.5 (3.0 - 4.0) |
| *P* for linear trend |  |  | 0.040 |
| High school or above |  |  |  |
| High income | 248 | 137482 | 1.8 (1.6 - 2.0) |
| Middle income | 288 | 117750 | 2.4 (2.2 - 2.7) |
| Low income | 196 | 69715 | 2.8 (2.4 - 3.2) |
| *P* for linear trend |  |  | <0.001 |
| Mortality due to other causes | |  |  |
| Less than high school |  |  |  |
| High income | 74 | 11698 | 6.3 (5.0 - 7.9) |
| Middle income | 287 | 44412 | 6.5 (5.7 - 7.3) |
| Low income | 416 | 61537 | 6.8 (6.1 - 7.4) |
| *P* for linear trend |  |  | 0.642 |
| High school or above |  |  |  |
| High income | 325 | 137482 | 2.4 (2.1 - 2.6) |
| Middle income | 507 | 117750 | 4.3 (3.9 - 4.7) |
| Low income | 369 | 69715 | 5.3 (4.8 - 5.9) |
| *P* for linear trend |  |  | <0.001 |

**
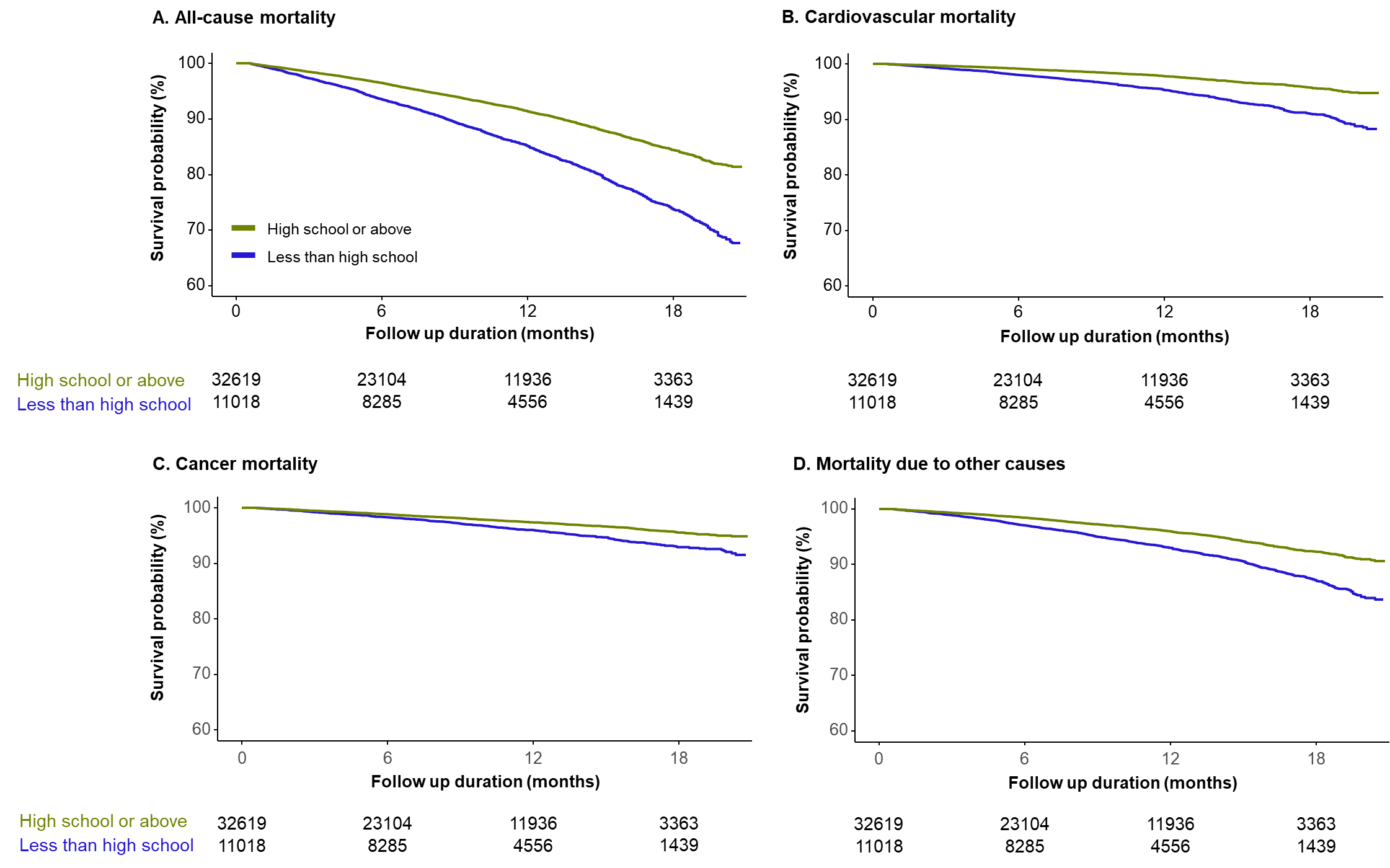
**

**Supplementary figure 1. Kaplan-Meier plots of time to all-cause and cause-specific mortality during follow-up by education level**

**
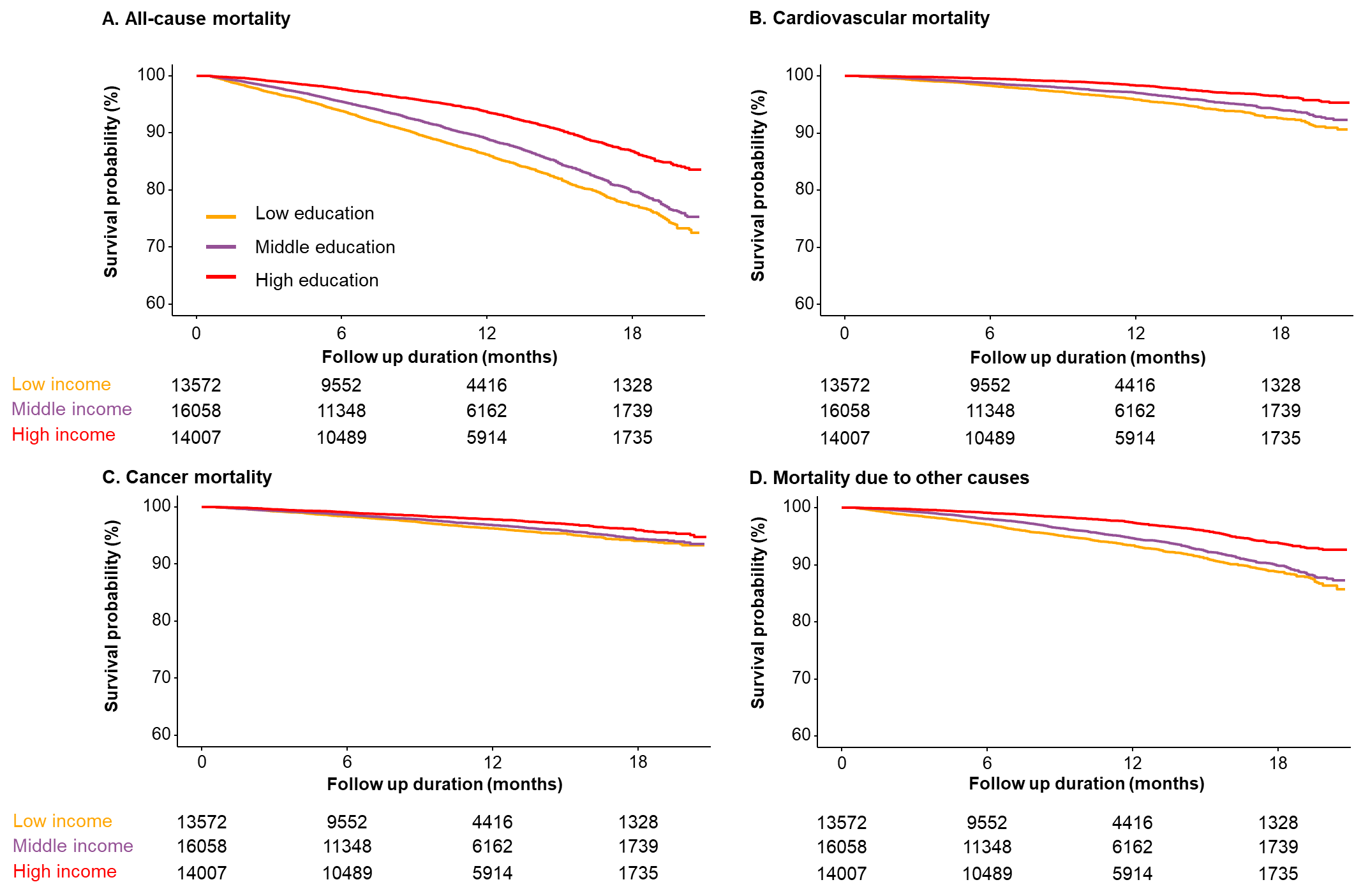
**

**Supplementary figure 2. Kaplan-Meier plots of time to all-cause and cause-specific mortality during follow-up by income level**
